# Improving quality control for in‐clinic hematology analyzers: Common myths and opportunities

**DOI:** 10.1111/vcp.13154

**Published:** 2022-09-12

**Authors:** Helen T. Michael, Mary B. Nabity, C. Guillermo Couto, Andreas Moritz, John W. Harvey, Dennis B. DeNicola, Jeremy M. Hammond

**Affiliations:** ^1^ IDEXX Laboratories, Inc Westbrook Maine USA; ^2^ Department of Veterinary Pathobiology College of Veterinary Medicine, Texas A&M University College Station Texas USA; ^3^ Couto Veterinary Consultants Hilliard Ohio USA; ^4^ Department of Veterinary Clinical Sciences Justus‐Liebig‐University Giessen Germany; ^5^ Department of Physiological Sciences, College of Veterinary Medicine University of Florida Gainesville Florida USA

A robust quality management system for automated hematology analyzers is crucial for generating high‐quality results and instilling confidence in analyzer function. A comprehensive quality management system comprises multiple aspects. Two major aspects are quality assurance (QA) and quality control (QC). QA is a framework and strategy for ensuring quality across the entire process, from blood draw through reported results. QA thus encompasses preanalytical to postanalytical variables related to result generation. QC, on the other hand, is focused exclusively on the analytical portion of quality management to ensure the function and performance of the analyzer itself. QC facilitates identification of analyzer problems so they can be addressed and fixed in a timely fashion. Recommendations for QC, including QC materials (QCMs), frequency of QC, and interpretation of QC results, are specific for the analyzer.

The ideal QCM would (1) be able to assess the function and performance of all aspects of the hematology analyzer, (2) have a long shelf life, (3) be easy for the user to run and interpret, and (4) instill confidence in results for all species tested. To understand these requirements, it is imperative to understand the workflow of an automated hematology analyzer. Although the specifics vary among analyzers, the workflow follows the same general pattern (Table 1). The recommended QCM and the algorithms to interpret the QC results vary among analyzers due to differences in chemistry and detection methods. In addition to basic analyzer function, QC for veterinary analyzers should be able to detect species‐specific differences in performance, including bias and drift.

**TABLE 1:** Preanalytical (operator) and analytical steps for analysis of hematology samples on in‐clinic hematology analyzers. Ideal QC methodologies should assess all aspects of analyzer function

Steps in sample analysis
Preanalytical
Patient preparation
Sample collection
(Sample storage)
Mixing
Analytical
Aspiration by analyzer
Analyzer chemistry (dilution, staining, lysis, etc)
Transport dilution to detector
Data collection
Algorithm for data interpretation
Result reporting

For analyzers in veterinary practices, ease of performing and interpreting QC is particularly important. Unlike reference or academic laboratories, staff in clinics typically do not have extensive training in quality management and may not have the same commitment to following QC recommendations and troubleshooting issues.^1^ Recommendations for in‐clinic analyzers are often similar to those for reference laboratory instrumentation but with less frequent QC analysis and simpler rules for interpretation. For example, manufacturer‐recommended QC frequency for in‐clinic analyzers is often once per month versus daily.

Regulations mandating QA and QC for veterinary in‐clinic analyzers vary regionally. Nevertheless, one study of in‐clinic analyzers in human medical settings, where stringent mandatory regulations exist,^2^ found that 19% of operators had not been trained to use the analyzer, 25% of operators failed to follow the manufacturer's procedures, and 32% failed to perform QC.^3^ The results of such a study would likely be similar or worse if done in veterinary practices. Practitioners often use in‐clinic analyzers to streamline patient care with rapid results that can immediately inform patient care, and they might not understand the importance of QC for instilling confidence in those results or the risks of not performing QC.

Hematology QC is often misunderstood by general practitioners, clinical pathologists, and other veterinary specialists. There are several myths surrounding QC that need clarification for better evaluation of the true benefits and shortcomings of traditional QC and QCM. In this editorial, we will address some common myths about QCM functionality and opportunities to continue to improve QC for in‐clinic hematology analyzers through automation and inclusion of patient samples.

## Myth #1: Quality control materials are representative of patient samples and contain all aspects of the reported differential cell count

Many practitioners and pathologists assume that fixed cell QCM (FC‐QCM) closely mirrors patient samples and contains red blood cells (RBCs), white blood cells (WBCs), and platelets. The use of fresh cells in commercial QCM is unfortunately impractical because their shelf life is only days. Therefore, traditional FC‐QCM uses cells that are mixed with a fixative (eg, glutaraldehyde) to improve stability and shelf life of the FC‐QCM.

The formulation and source of cells in commercial FC‐QCM are proprietary, and there is limited information available to practitioners or clinical pathologists. Publicly available information about FC‐QCM is often vague and includes statements like “partially derived from human sources.”^a^ Cells in the FC‐QCM can come from mammals (usually human), reptiles, birds, or a combination thereof. In some cases, FC‐QCM use nucleated erythrocytes from reptiles or birds as WBC surrogates to assess WBC parameters since avian and reptile erythrocytes are easy to obtain in large numbers. Some commercial FC‐QCM use small erythrocytes to assess analyzer platelet identification. When mammalian cells are used, they are usually human and may not accurately represent performance on veterinary species. Due to the lack of transparency about formulation, the true contents of a commercial FC‐QCMs are not known, but they are not necessarily a close mimic of veterinary patient samples.

**FIGURE 1:**
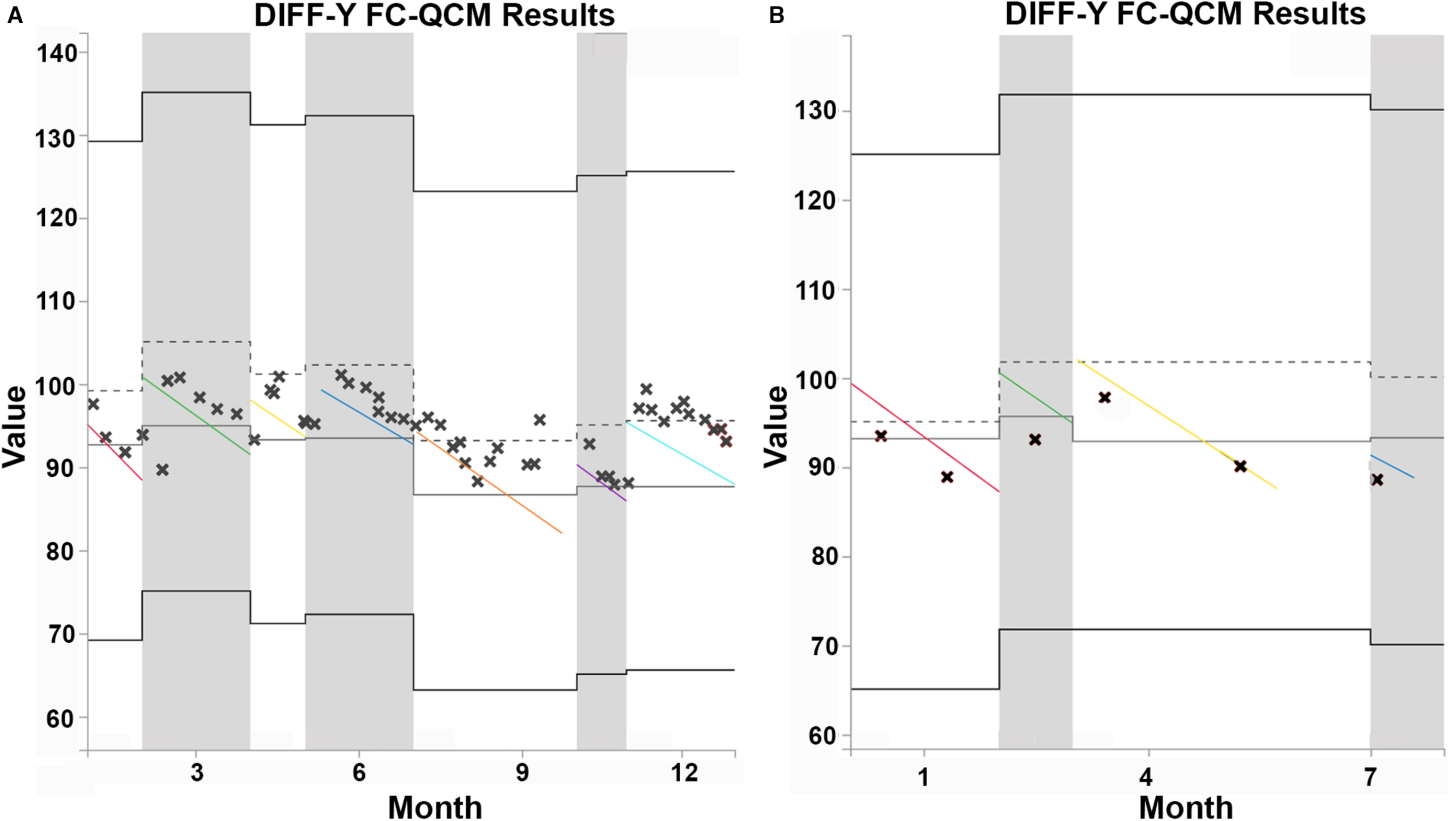
DIFF‐Y control plots from two different clinics showing expected changes over time in the fixed cell quality control material (FC‐QCM targets). DIFF‐Y is a standardized parameter identifying the average position of a cell population on the *Y*‐axis on the IDEXX ProCyte Dx. Quality control (QC) results over time are shown for a clinic performing daily to weekly QC (A) and a clinic performing monthly QC (B). Diagonal lines show the expected change in the QC target with aging for each lot. Central lines indicate the recommended target for the QC lot at the time of manufacturing (dotted) and the field population response (solid). Bold outer lines represent ± standard deviations from the target. Lots are delineated by alternating gray and white backgrounds.

Even with fixation, the shelf life of FC‐QCM is short. FC‐QCMs are often exquisitely sensitive to temperature, and improper storage further reduces their shelf life. Required FC‐QCM for the Abaxis Vetscan HM5 hematology analyzer, for example, indicates that it is usable after opening for “up to 14 days if it is properly stored.”* Even when properly stored, changes in FC‐QCM can occur over time due to aging. For some parameters, the expected aging changes in the QCM lead to alterations in QC targets over the lifespan of the QCM (Figure 1). Mishandling of the lot or using it beyond its expiration date causes further degradation of cells and may affect the utility of the QCM to provide accurate information about analyzer function. The exact QC targets and ranges can vary between lots of FC‐QCM. It is recommended to update the analyzer with the QC targets for the new lot and to analyze both the new and old lots when switching lots to understand lot‐to‐lot bias. If clinics use FC‐QCM every 2–4 weeks according to analyzer manufacturers' recommendations, they may only use a single lot of FC‐QCM for one or two QC analyses due to the short shelf life. This makes it burdensome for clinics to compare lots and set lot‐specific QC targets. However, skipping this step diminishes the ability of the FC‐QCM to assess analyzer function and drift over time. The short shelf life of the FC‐QCM, therefore, creates a significant financial and logistical burden on veterinary clinics and can prevent clinics from realizing the full potential value of QC.

## Myth #2: Fixed cell quality control material is handled by the analyzer like patient samples

In addition to differences between FC‐QCM components and patient samples, there are fundamental differences in analyzer workflow for FC‐QCM and patient samples. Many of the steps and algorithms are QCM specific (Table 2), which impairs the ability of FC‐QCM to fully evaluate analyzer function. The differences begin with sample handling in the clinic before analysis. FC‐QCM must be stored in the refrigerator, brought to room temperature before use, and then quickly returned to the refrigerator. They are also mixed and loaded onto the analyzer differently than patient samples. Fixation of cells leads to alterations in stain uptake by cells, cell lysis, and response to other reagent chemistry. FC‐QCM, therefore, may not be able to identify analyzer problems with sample loading, stains, and other reagent chemistry.

**TABLE 2:** Similarities and differences in the workflow for patient samples and quality control materials (QCMs) used in different quality control strategies

Step	Fixed cell QCM	Bead‐based QCM	Patient‐based population analysis
Preanalytical			
Sample collection	Obtained from manufacturer	Obtained from manufacturer	**✓**
Sample handling	QCM specific	QCM specific	**✓**
Sample loading	QCM specific	QCM specific	**✓**
Analytical			
Aspiration	**✓**	QCM specific	**✓**
Analyzer chemistry	QCM‐specific	NA	**✓**
Aspiration	**✓**	**✓**	**✓**
Fluidics	**✓**	**✓**	**✓**
Event detection	**✓**	**✓**	**✓**
Event classification	QCM specific	QCM specific	**✓**
Report generation	QCM specific	QCM specific	**✓**
QC interpretation	QC specific	QC specific	QC specific

Analyzers can extrapolate information from standardized materials to evaluate individual functions and do not need QCM to mimic patient samples. As we discussed before, FC‐QCM can evaluate many aspects of analyzer function despite differences from fresh patient samples. QC‐specific algorithms use standardized materials to compare current with expected analyzer function and extrapolate that information to analyzer performance on patient samples. For example, comparing detected events to expected events for standardized QCM concentration allows evaluation of the dilution, mixing, and fluidics functions. Moreover, evaluating changes in event size and complexity for standardized QCM provides information about sensor and laser alignment. Thus, evaluation of standardized materials is able to instill confidence that analyzer function is appropriate.

Different types of analyzers (eg, impedance analyzers, flow cytometry analyzers, and fluorescence flow cytometry analyzers) function differently and have different required features for QCM. Commercial FC‐QCM may be marketed specifically for one analyzer or for use with multiple analyzers. For instance, Para 12 Extend (Streck) is marketed for analyzers made by eight different companies, but analyzer‐specific QC targets and reference intervals are provided to meet the needs of each analyzer. However, the applicability of one FC‐QCM for different analyzers depends on if the formulation provides information that is relevant to that analyzer's technology and methodology. Analyzer algorithms and targets must be developed for the specific recommended FC‐QCM since there are differences between analyzer requirements and FC‐QCM components.

**FIGURE 2:**
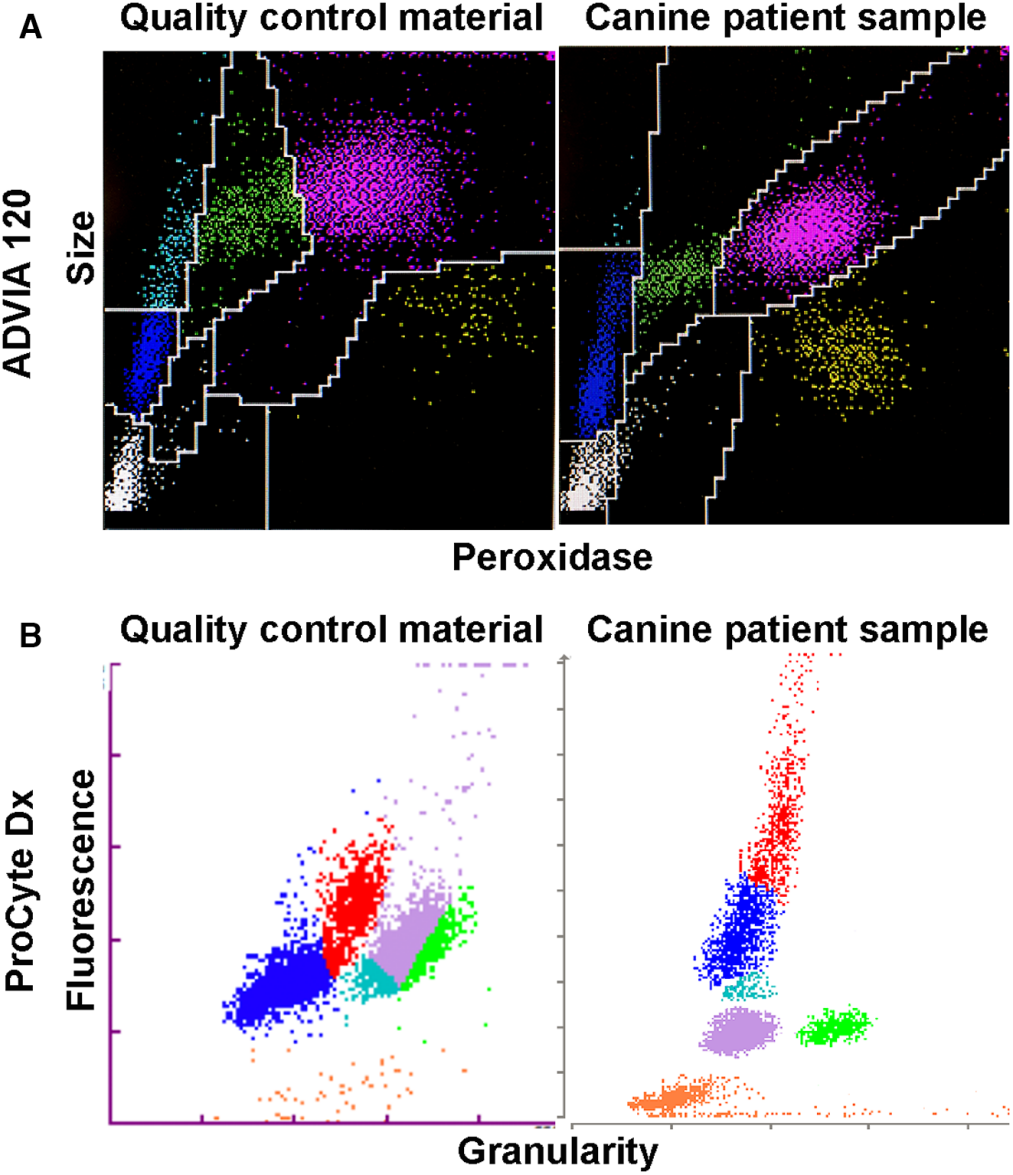
Scatterplots from the ADVIA 2120 peroxidase channel and WBC plot from the IDEXX ProCyte Dx of the recommended quality control material (QCM) for each analyzer and normal canine samples. (A) ADVIA 2120 QCM and canine scatterplot differences are on par with interspecies differences. (B) ProCyte Dx scatterplots for QCM are dissimilar from canine or other patient scatterplots. Both QCMs provide standardized evaluation of analyzer function and instill confidence in the results of their respective analyzers. Similarity of QCM to patient samples is not predictive of whether the QCM is able to evaluate analyzer functions.

Analyzer algorithms for identifying cells (eg, size, complexity, fluorescence) are species‐specific and result in different scatterplots. Similarly, FC‐QCM scatterplots do not necessarily mimic the plots for any veterinary species. Scatterplots for both patient samples and FC‐QCM vary among analyzers because analyzers identify and characterize cells using different characteristics. If the recommended FC‐QCM for the ADVIA 2100/120 is run through the analyzer as a sample, it looks reasonably like canine patient samples. When the same thing is done with the recommended FC‐QCM on the IDEXX ProCyte Dx analyzer, plots look less like their corresponding canine patient scatterplots (Figure 2). Although these differences can be surprising or jarring for human observers, the similarities or differences between patient and FC‐QCM plots are not informative about whether the FC‐QCM is adequate for providing the needed information for the QC‐related analyzer algorithms.

## Myth #3: QC results reliably indicate that patient results are acceptable and correct

As noted above, FC‐QCM differs from veterinary patient samples. Most of us are familiar with the changes to staining patterns with Wright‐type stains due to formalin exposure or fixation, but fixation of any type can alter fluorescence, staining characteristics, and response to chemical lysis. As a result, FC‐QCM cannot reliably identify changes in analyzer chemistry that could impact patient samples from one or more veterinary species.

**FIGURE 3:**
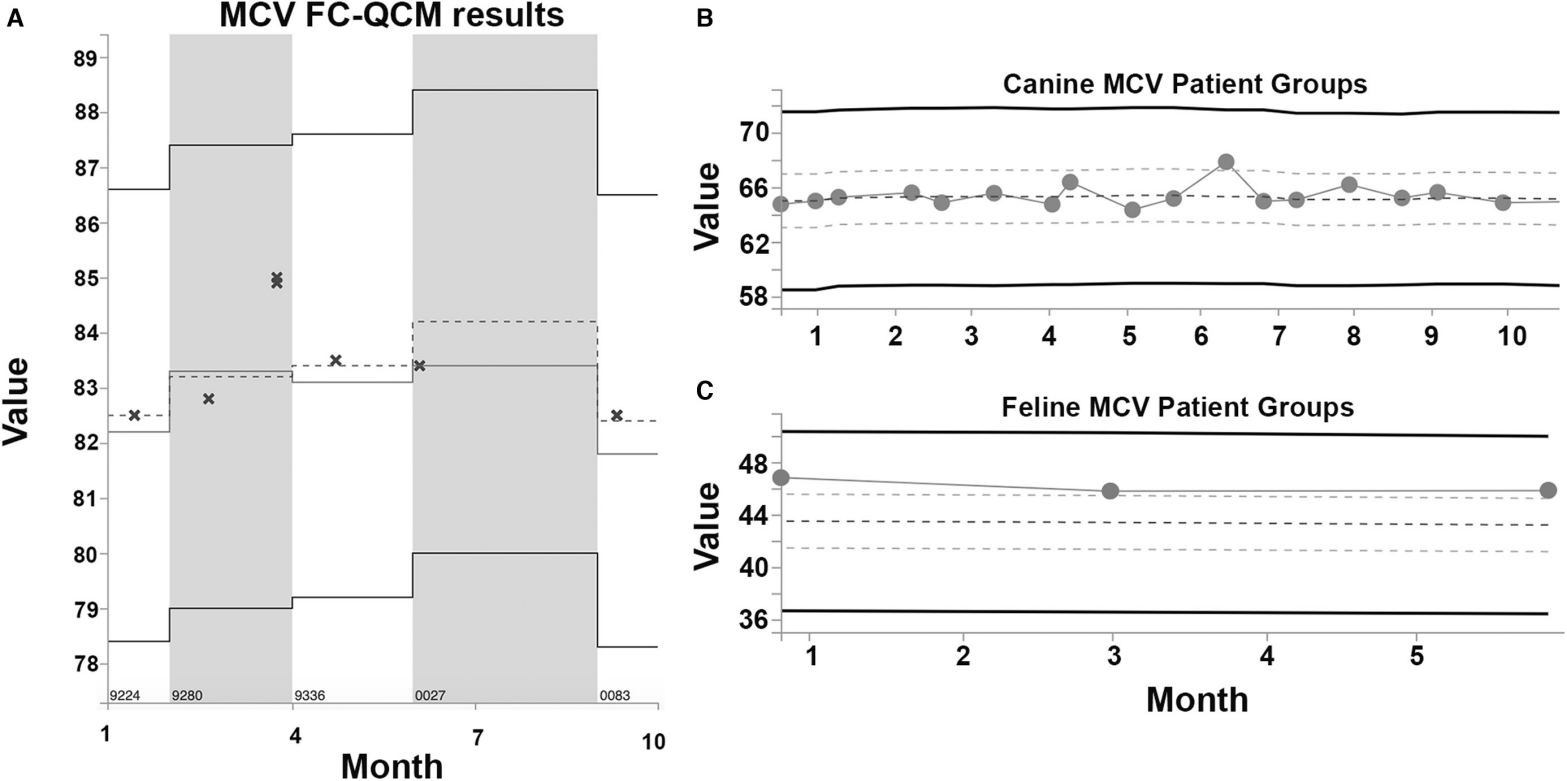
Example of an instance of species‐specific bias in patient‐based population analysis quality control (PBA‐QC) means despite acceptable fixed cell QC results. (A) FC‐QC results fall close to the QC target for MCV (central dotted line) and field population response (central solid line). Bold outer solid lines show ±3 standard deviations (SDs). Gray and white shaded areas FC‐QC material lots. (B, C) PBA‐QC patient group dots represent the mean of 10 patient samples. Canine samples trend near the median for all ProCyte Dx analyzers (center dashed line), while feline patients are routinely above the mean and at or above 3 SDs above the mean (dotted lines).

**FIGURE 4:**
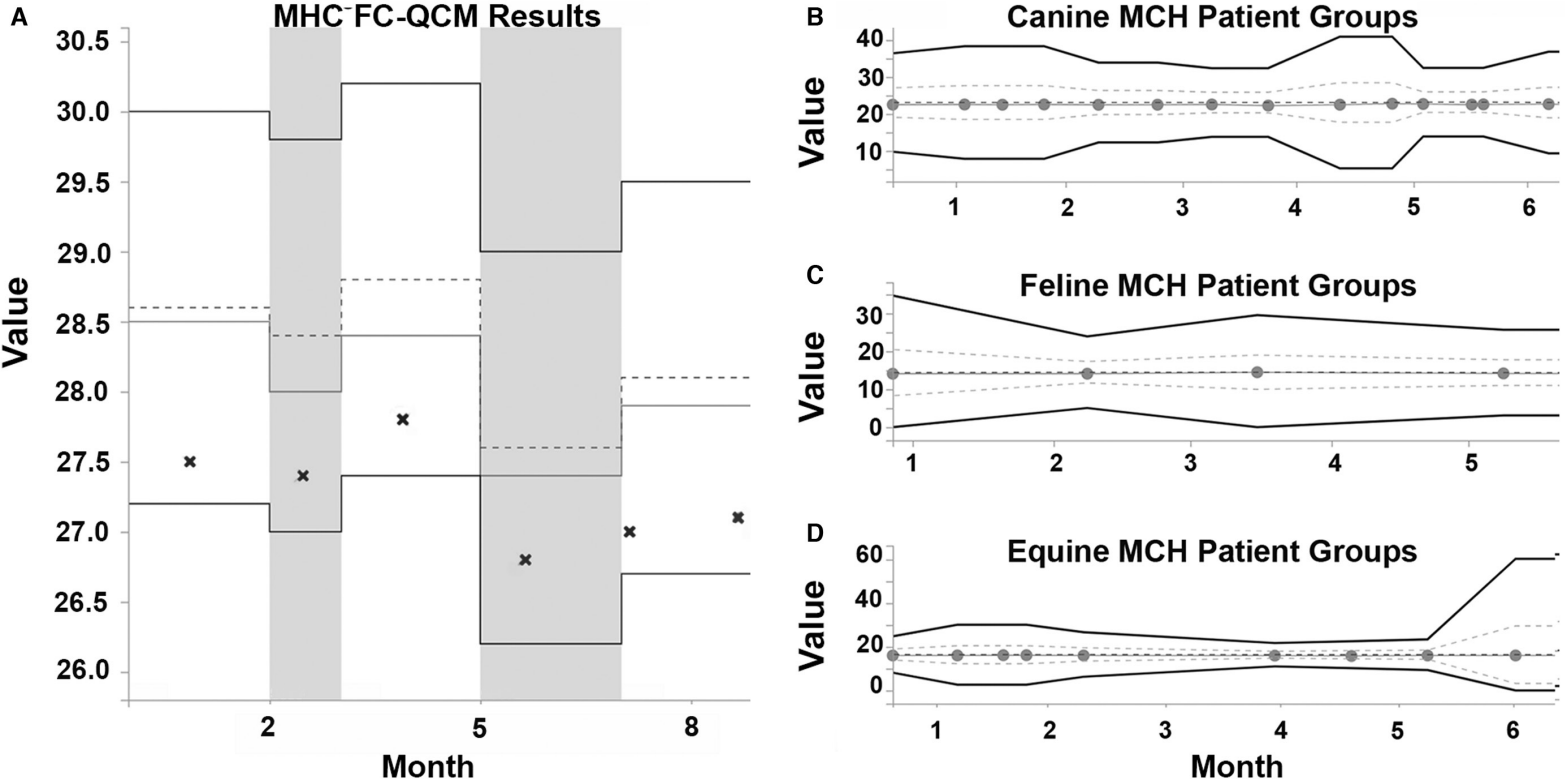
Example of an analyzer with fixed cell (FC) quality control (QC) materials results tracking consistently below the QC target. (A) FC‐QC results are reliably near below the QC target (dotted line) and field population response (center solid line) for each lot but within 3 standard deviations (SDs) of the target (outer bold solid lines). The QC target for MCH (central dotted line) and field population response (central solid line). Bold outer solid lines show ±3 standard deviations (SDs). These results would pass a single rule QC interpretation but may fail multi‐rule interpretations. (B–D) Patient‐based population analysis QC uses the mean of groups of 10 patients (dots). Means for patient groups fall on the expected means (center dotted line) for canine, feline, and equine results across all analyzers.

Chemical alterations or degradation can have variable effects on different species. For example, minor osmolality changes in analyzer chemistry may not affect lysis of easily lysed canine RBCs but might reduce lysis of more lysis‐resistant feline and equine RBCs.^1^ Changes in analyzer chemistry can also cause bias affecting one or more veterinary species but not FC‐QCM. As a result, QC results from FC‐QCM may indicate acceptable analyzer function even though results for some species are affected (Figure 3). Conversely, clinicians might erroneously believe that drift in the FC‐QCM results indicates that patient results will be incorrect (Figure 4). QC results provide information about the performance of an analyzer on standardized QCM but should not be interpreted to always reflect whether patient results are reliable. Recognition of factors or changes that differentially affect FC‐QCM results and veterinary patient results can be difficult if only one type of QC evaluation is performed. Continued improvement in QC recommendations to more quickly and accurately identify the problems that only affect patient samples is desirable and would facilitate appropriate corrective actions.

## Myth #4: In‐clinic analyzer QC frequency and interpretation should mimic reference laboratory QC frequency

There are required minimum QC requirements for reference and academic laboratories, but the actual QC frequency is often adapted to meet the risk analysis for the laboratory. This can lead to substantial differences among laboratories in the frequency of QC and in the choice of statistical rules chosen for QC interpretation. One study surveying laboratories at large, well‐respected academic medical centers testing human patient samples found “no systematic approach to defining QC rules or frequency.”^4^ QC frequency varied from every 2 hours to every 24 hours, and selection of QC rules was often through institutional experience rather than through adoption of evidence‐based rules like “Westgard Rules.”^4^ This variability in approaches to QC frequency and interpretation is likely similar in veterinary academic settings and amplified in veterinary clinics where there are fewer regulations and staff is less educated on QC.

Risk assessment for choosing QC frequency and statistical analysis ideally includes identifying what potential problems could arise, the required result quality, risk to patient results, and what can be done to mitigate those risks.^5^, ^6^ In reference or academic laboratories, strategies like Six Sigma can be used to evaluate these risks and determine the optimal frequency of QC, the number of samples that can be analyzed between QC analysis, and the optimal rules for the interpretation of QC results^7^, ^8^, ^9^; however, many of these methods are impractical for use with in‐clinic analyzers in the general practice setting. Although the same types of analyzer problems can occur in the reference or academic laboratory and the in‐clinic laboratory setting, there may be differences in the quality goals, number of samples at risk, potential mitigating factors, and potential cost of mitigation. Manufacturers of in‐clinic hematology analyzers may recommend a minimum of weekly or monthly external QC instead of daily. These are meant as minimum recommendations that would be appropriate for clinics that analyze a moderate number of samples; however, they might not be optimal for clinics at the extremes who either analyze large numbers of samples or rarely use the analyzer. In many clinics, the ratio of FC‐QCM to patient sample analyses will be higher than in reference laboratories, even when minimum recommendations for QC are followed. If daily external QC is performed, this ratio would be further increased to a point that the time and financial burden could inadvertently discourage QC compliance for clinics. QC recommendations for in‐clinic analyzers would ideally incorporate more specific risk assessment for different use scenarios to help clinics understand the risk‐benefit analysis for their circumstances.

**FIGURE 5:**
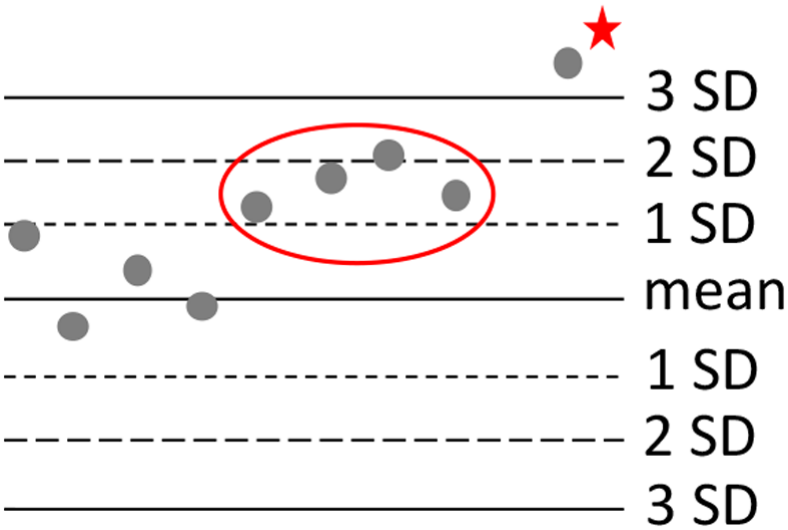
Schematic of a Levy‐Jennings plot of simulated QC results showing how interpretation of the QC results would vary depending on which Westgard Rule was used for statistical interpretation. If a 1_3S_ rule was used, the results would be considered if one result was more than 3 standard deviations (SDs) from the mean. The first unacceptable QC result would be the result marked with a red star. If a 4_1S_ rule was used, four results more than 1 SD from the mean would be unacceptable (red oval). Using a 4_1S_ rule would identify analyzer problems at an earlier time in this example than a 1_3S_ rule. Choice of statistical analysis rules should be based on risk analysis and balanced between risks of false rejection and false acceptance of QC results.

Choice of statistical methods to interpret QC results is part of the risk analysis. A variety of statistical methods can be used for the interpretation of QC results with different degrees of rigor for identifying analyzer issues. Reference laboratories and clinics will have different ideal balances of maximizing the potential to identify analyzer issues and minimizing unnecessary troubleshooting and false rejection of results. Levey‐Jennings charts are commonly used to track performance of QC over time.^10^, ^11^ These charts are often generated by the analyzer for easy viewing to assess QC performance. Different rules exist to evaluate whether the QC results are acceptable in different situations, and “Westgard Rules” are most commonly used.^12^ These rules vary in complexity from single rule to multi‐rule methods and have different levels of stringency and different balances of risk (Figure 5). Most manufacturers recommend a single rule method, primarily the 1_3S_ rule. Under the 1_3S_ rule, results are not acceptable if they are more than 3 standard deviations (SDs) from the mean or target.^13^ Target ranges for this rule are fairly wide and minimize false rejection of QC results. Single rules can be tightened to be more sensitive to smaller drift and reject results with smaller deviations, for example, by incorporating both the magnitude and frequency of a deviation to determine if a QC result is acceptable. A 4_1S_ rule, for instance, would reject a 1 SD if it was the fourth such deviation in a row. A 4_1S_ rule would be more stringent and detect smaller deviations from the target but would be more likely to falsely reject QC results. Multiple rules (ie,1_3S_ and 4_1S_) can be used to incorporate a variety of different magnitudes and durations of risk where any included rule can be used to reject a result. Similar to the frequency of QC, the choice of rule needs to reflect the particular risk management strategy appropriate for the reference laboratory or clinic, the volume of patient samples, and frequency of QC analyses. As such, complex rules are appropriate for reference laboratories with high sample volume and trained personnel but are unnecessarily complex for use in clinics. The frequent false rejection of QC results in clinics leads to unnecessary and frustrating analyzer downtime and troubleshooting that could erode the clinic's commitment to following QC recommendations.

## OPPORTUNITIES FOR IMPROVING THE FUTURE OF QC

These common QC myths hide some of the true opportunities for improvement in QC, particularly QC in the in‐clinic setting, where improvements in ease and compliance could lead to improved confidence in results from hematology analyzers. Although FC‐QCM provides many of the answers about analyzer function and performance, FC‐QCM alone does not allow us to assess all the components. Technological advances and research have resulted in new QC methodologies and materials being used in pockets across the industry without broad adoption. Many of these advances offer opportunities for continued improvement in hematology QC by combining different strategies and new QCM to provide a better overall view of analyzer function and thereby improve the potential for identification and resolution of analyzer issues.

### Opportunity #1: Use alternate noncellular QCM to enhance shelf life and stability of QCM

Noncellular QCMs, including manufactured beads, have many of the benefits of FC‐QCM and have much longer stability and shelf life. Bead‐based QCMs and calibration materials are commonly used as standards to verify fluidics, fluorescence, and optical performance in flow cytometers.^14^, ^15^ Bead‐based QCM can be manufactured to individual specifications with high reproducibility. The design and manufacture of beads allow for a wide variety of bead types that can be specialized to best evaluate the analyzer function. The design of bead‐based QCM can be specific to the cellular features that the analyzer evaluates, including size, refractility, and fluorescence. More complex features such as granularity or surface textures could be generated if relevant to the analyzer. The bead design can include features that allow the same beads to evaluate analyzer functions for red cells, white cells, and platelets, or for beads to specifically represent a single cell type.

Some in‐clinic hematology analyzers, like the IDEXX LaserCyte DX analyzer, already use bead‐based QCM. Qualibeads are run with each sample to ensure that the analyzer functions properly and is standardized within and across runs on this analyzer.† Bead‐based QCMs, like FC‐QCMs, do not directly mimic patient sample cells and require QCM‐specific algorithms and workflow steps (Table 2). As such, neither bead‐based QCMs nor FC‐QCMs assess all components of analyzer functionality on patient samples. However, the longer stability and easier storage of bead‐based QCM make them more appealing for use on in‐clinic analyzers. Many bead‐based QCMs can be stored at room temperature and do not degrade with higher or lower temperatures. This makes bead‐based QCM easier to ship and store in regions with temperature extremes, and it simplifies QC workflow. Improved thermal stability and shelf life also allow some bead‐based QCM to be stored in the analyzer itself, thereby facilitating automation of QC analysis to reduce the clinic workflow and improve compliance with QC recommendations.

### Opportunity #2: Incorporate patient samples into QC to better model patient workflow and ensure patient results are acceptable

Integration of patient samples into the QC strategy can help to fill some of the gaps left by FC‐QCM or bead‐based QCM. QC uses patient samples and can identify species‐specific issues related to analyzer chemistry that are missed by other QCM. Several methods using patient samples have been described and found to be both practical and successful at evaluating the function of veterinary hematology analyzers.^16^, ^17^, ^18^ These strategies each have their own benefits and drawbacks.

Patient‐based population analysis quality control (PBA‐QC) uses populations of patients previously analyzed on the instrument to identify drift, species‐specific bias, or problems with analyzer chemistry. Since it uses data from actual patient samples, these data are generated with the normal patient workflow and can be used to assess all steps of the analysis, including preanalytical sample handling. Weighted moving averages from patient samples have been used to monitor analyzer performance for over 45 years for human hematology analyzers.^19^, ^20^, ^21^, ^22^ Using averages allows both normal and abnormal patient results to be included in the QC analysis. However, clinics that analyze primarily or only abnormal samples may have averages that deviate from the normal targets. On the IDEXX ProCyte Dx, an in‐clinic analyzer that uses PBA‐QC, weighted average analysis evaluates groups of the most recent 10 patient sample results (Figures 3B–D and 4B–D). Since grouping and analysis can be performed automatically during analyzer downtime or in the background, PBA‐QC does not require additional effort from the clinics to perform or seek out QC results. The frequency of PBA‐QC analysis reflects the volume of patient samples analyzed and is, therefore, adaptive to the individual clinical setting and proportional to clinic analyzer use. Comparisons can also be made between the results from one individual clinic and the results of all clinics using the analyzer. This can provide additional information about drift for analyzers in both low‐volume and high‐volume clinics.

Repeat patient testing (RPT‐QC) uses patient samples differently and has been investigated in both human and veterinary medicine.^17^, ^23^ RPT‐QC uses the expected rate of subtle changes in patient samples over time to evaluate analyzer performance.^17^, ^24^ Samples are re‐processed at specific intervals after the original analysis to look for variation in samples. Algorithms are used to compare the expected results at different sample ages to the actual recorded values to identify both random and systematic error in the analyzer. However, since each sample can only be stored for approximately 24 hours, RPT‐QC requires continual selection of new patient samples for QC. Given the requirement for daily identification of QC samples, careful storage, and repeat analysis at specific timepoints, RPT‐QC is feasible for reference laboratories and possibly for in‐clinic analyzers from some high‐volume clinics, but not for most clinics where sample availability, cost, and technician training and time would provide major hurdles.

Strategies for using patient samples have different strengths and drawbacks from FC‐QCM and bead‐based QCM. Since patient samples are used, there are no QC‐specific analyzer workflows (Table 3), and species‐specific drift can be detected for any species included in QC analysis. However, patient samples alone lack the standardization that FC‐QCM allows and require access to normal samples. PBA‐QC removes most logistical burden of FC‐QCM and bead‐based QC for in‐clinic analyzers. Integration of traditional QCM and patient‐based analyses within the clinic or analyzer QC strategy can provide the benefits of each approach while filling in the gaps from the use of any single approach (Table 4). Standardized QCM provides specific information about analyzer function and calibration, and patient‐based analysis provides information about performance on chemistry function and individual species. PBA‐QC can run in the background on the analyzer and identify issues in patient sample workflow missed by QCM analysis or that occur between routine scheduled QCM analysis.

**TABLE 3:** Storage requirements, additional workflow requirements, and targets for different types of quality control materials (QCMs) used in different quality control strategies

	Fixed cell QCM	Bead‐based QCM	Patient‐based population analysis
Materials	Proprietary mix of mammalian +/− nonmammalian cells	Manufactured beads	Patient samples
Reagent storage	Refrigerated—bring to room temperature for use	Room temperature	NA
QCM shelf life	<3 months	>6 months	NA
Type of QC run	Manual QC	Automatic or manual QC	NA
Target comparison	Compare to QCM targets	Compare to QCM targets	Population statistics

**TABLE 4:** Alignment of quality control materials and different quality control strategies with the ideal characteristics of a material or strategy

Ideal characteristics	Fixed cell QCM	Bead‐based QCM	Patient‐based population analysis
Information on all aspects of analyzer function	✗	✗	**✓**
Long shelf life	✗	**✓**	N/A
Potential for automation	✗	**✓**	**✓**
Ability to assess function across species	✗	✗	**✓**
Standardized QC materials	**✓**	**✓**	✗

### Opportunity #3: Automate QC to improve compliance with QC recommendations and interpretation

Traditionally, quality evaluation and maintenance of in‐clinic analyzers have been entirely the responsibility of the clinic staff. However, there is wide variability in the commitment of clinics to perform QC and to understand the importance and significance of QC and quality management. There can also be logistical hurdles for clinics due to high patient volume and staff turnover. Automation of QC provides a path around these hurdles, presenting an opportunity to improve confidence in results from in‐clinic analyzers across all clinics and to ensure that QC is performed at the recommended minimum frequency.

Automated QC can be performed by the analyzer outside of work hours and without requiring effort or commitment from clinic staff. Additionally, automation of QC interpretation would improve and standardize recognition of analyzer problems and assist in troubleshooting for in‐clinic analyzers. Centralization of QC interpretation could also more easily identify drift or other subtle issues by comparing an analyzer in one clinic to the general population of all similar analyzers. These subtle problems could be centrally identified with notifications automatically sent to clinic staff with appropriate next steps. Additionally, QC could automatically be repeated if there was a run failure, and analyzer or calibration issues could be fixed remotely. Partially transferring responsibility for performing QC analysis from the clinic to a centralized, automated system would improve overall compliance with QC recommendations and decrease strain on clinics. Automated QC could also facilitate using more stringent interpretive rules by removing resource and time barriers for more stringent analyses from the clinic. Automating both the QC and its interpretation should improve analyzer performance and instill better confidence in results from in‐clinic hematology analyzers.

## SUMMARY

Despite the importance of QC, it is often misunderstood or even ignored by clinicians. There are pervasive misconceptions and myths about quality control, the nature and requirements of QCM, and QC for in‐clinic hematology analyzers. While FC‐QCM provides valuable information about the health and performance of the analyzer system, they do not directly mimic patient samples and patient workflow, and they have a short shelf life. This short shelf life can be financially and logistically burdensome for clinics with low sample numbers and infrequent QC analysis. Direct mimicry of patient samples is not necessary for the benefits associated with QCM. Noncellular QCM can have similar benefits without the short shelf life of FC‐QCM. However, both cellular and noncellular QCM fail to assess all aspects of patient sample analysis. Combining QCM and patient sample analysis (eg, PBA‐QCM) can provide more ideal QC information about the analyzer function and identify species‐specific issues with analyzer chemistry or drift.

Risk assessment for in‐clinic analyzers is different than for reference laboratory analyzers. As a result, the QC frequency and analysis recommended for the laboratory analyzer might not be appropriate for many clinics, especially ones with low sample numbers. There are unique challenges to ensuring appropriate analyzer function for in‐clinic analyzers and QC compliance. Automation of QC analysis can help to improve compliance with minimum QC recommendations and to ensure accurate results for in‐clinic analysis. This automation can also relieve some of the burden of QC from busy clinics.

There are opportunities to continually improve the QC experience and compliance for in‐clinic analyzers by extending QCM shelf life, improving thermal stability of QCM, and automating many of the QC activities. The concepts described here can be adapted and applied to different hematology analyzer technologies and are not specific to any particular manufacturer. Hematology analyzer manufacturers should strive toward improving QC in ways that increase the quality of results from in‐clinic analyzers, reduce the financial and logistical burdens of QC, and improve overall patient care by minimizing analytical errors.

## CONFLICT OF INTEREST

JH, HTM, and DBD are or have been full‐time employees of IDEXX Laboratories, Inc. All other authors serve as consultants for IDEXX Laboratories, Inc.
